# Intranasal dexmedetomidine vs. nitrous oxide for procedural sedation in juvenile idiopathic arthritis (INDEXJIA): a randomized crossover clinical trial

**DOI:** 10.1007/s00431-026-07259-w

**Published:** 2026-07-21

**Authors:** Miikka Tervonen, Outi Peltoniemi, Paula Vähäsalo, Paula Keskitalo, Sirja Sard, Tytti Pokka, Merja Kallio

**Affiliations:** 1 Department of Children and Adolescents, Oulu University Hospital, P.O. Box 23, 90220 Oulu, Finland; 2 Research Unit of Clinical Medicine, Medical Research Center Oulu, University of Oulu, Oulu University Hospital, Oulu, Finland; 3 Helsinki New Children’s Hospital, HUS and University of Helsinki, Stenbäckinkatu 9, Helsinki, 00290 Finland

**Keywords:** Child, Intra-articular injection, Pain, Procedural sedation, Rheumatic disease

## Abstract

This study aims to compare intranasal dexmedetomidine with inhaled nitrous oxide for procedural pain relief and sedation during intra-articular injections in a pediatric rheumatology outpatient clinic. Patients from 1 to 18 years-of-age requiring 1–5 intra-articular injections were randomized to receive 2 µg/kg intranasal dexmedetomidine or inhaled nitrous oxide in the first visit. When the patient required subsequent treatment visit, the other sedative was administered. The primary outcome was patient-reported pain immediately after recovery from sedation. Hemodynamic and respiratory parameters, level of sedation and patient’s pain assessed by the parent, rheumatologist, and nurse were secondary outcomes. Out of 119 eligible patients, 68 were included, and 43 received both medications at two separate visits. Overall pain experience was mild and did not differ between dexmedetomidine and nitrous oxide in patient-reported pain immediately after the procedure (difference, 0.2; 95% CI − 0.7 to 1.2, *p* = 0.65) but the patients recalled milder pain after nitrous oxide in the follow-up survey (difference, − 1.3; 95% CI − 2.3 to − 0.3; *p* = 0.01). Milder pain was reported during the second visit compared to the first visit regardless of the sedative used (difference, − 1.2; 95% CI − 2.1 to − 0.2; *p* = 0.02). In the follow-up survey, majority (69%) of patients would prefer nitrous oxide to dexmedetomidine for sedation in future.

*Conclusions*: Both intranasal dexmedetomidine and inhaled nitrous oxide offered effective analgosedation for intra-articular injections in children. Nitrous oxide resulted in better patient experience and became their preference for future sedation.

Trial registration: ClinicalTrials.gov TRN: NCT03069638. https://clinicaltrials.gov/study/NCT03069638. The date of registration 2016–12-20.

**Table:** 

**What is known:** • *Children suffering juvenile idiopathic arthritis often require repeated intra-articular injections.* •* Procedural sedation is being used during these procedures.*	
**What is New:** • *When intranasal dexmedetomidine and nitrous oxide was compared in a crossover design both agents provided effective analgosedation without difference in patient-reported pain immediately after the procedure.* • *Despite this, most patients favored nitrous oxide for future sedation.*

**Supplementary Information:**

The online version contains supplementary material available at 10.1007/s00431-026-07259-w.

## Introduction

Juvenile idiopathic arthritis (JIA) is the most common childhood rheumatic disease with a prevalence of 16–150 cases per 100,000 children in high-income countries [1]. Intra-articular corticosteroid injections (IACI) are commonly used to relieve acute arthritis, providing effective treatment with minimal systemic side effects [2–4]. Even successful IACIs can cause pain and fear in children, so procedural sedation is considered standard of care [5–7]. Minimal sedation with nitrous oxide combined with other pain relieving-agents has been shown efficient, safe and cost-effective for procedural sedation during IACI in pediatric patients [8–12].

Nitrous oxide (N₂O) is an inhalation anesthetic having both analgesic and sedative properties, rapid onset and elimination half-life of 5 min, making it favorable sedative agent for short procedures [13]. Dexmedetomidine (DEX) is a selective α_2_-agonist providing analgosedation and being commonly used for sedation during imaging studies in pediatric patients [14–16]. I.n. DEX sedation for minor painful procedures in pediatric emergency clinic have shown promise and doses up to 4 µg/kg have been reported safe in children [15, 17–19]. Despite the widespread use of N₂O sedation for pediatric IACI, i.n. DEX has not yet been investigated in this context. I.n. DEX might enable these procedures to be performed during routine outpatient visits without requiring dedicated gas delivery equipment or gas removal.

In this randomized crossover clinical trial, i.n. DEX was compared with N_2_O for procedural analgosedation in pediatric JIA patients. In addition, the safety profiles of both sedatives were assessed.

## Methods

This academic, prespecified, unblinded, randomized crossover clinical trial was conducted at the pediatric outpatient clinic of Oulu University Hospital, Finland, from February 1, 2017, to December 18, 2023. Patients were randomized to receive either N₂O or DEX at the first visit, and if a second treatment visit with IACI was needed, they received the alternate sedative. The trial was conducted in accordance with the principles of Good Clinical Practice guidelines and the Consolidated Standards of Reporting Trials (CONSORT) reporting guideline. The study protocol was approved by National Committee on Medical Research Ethics (Tukija) on May 20, 2016 (ID: 87/06.00.00/2016), the ethical committee of the Northern Ostrobothnia Health care district in October 19, 2016 (ID: 82/2016) and The Finnish Medicines Agency (Fimea) November 7, 2016 (ID: 2016–002065-66). Clinicaltrials.gov ID for this trial was NCT03069638.

The study subjects were eligible for enrollment in the trial if they had 1–5 inflamed joints requiring IACI at the time, were from 1 to 18 years of age, and were treated at the pediatric rheumatology outpatient clinic. Patients with severe congenital heart disease, severe obesity, severe neurologic condition, or moderate to severe sleep apnea were excluded. Eligibility was screened by reviewing the medical records. Patients’ medical history and current well-being were discussed with the accompanying parent to exclude contraindications for the study drugs. Electrocardiogram (ECG) was monitored before administration of sedation to rule out second or third-degree atrioventricular block, which was considered contraindication for DEX. Parents or legal guardians gave their written informed consent before participation. Patients aged 6 to 18 years gave their own written consent in addition to parental consent.

### Treatment

Using a computer-generated allocation sequence, patients were randomly assigned to receive either DEX or N_2_O for sedation during IACI at the initial treatment visit, with a crossover to the alternate sedative at the second visit. The allocation was concealed in sealed, numbered, opaque envelopes, which contained the name of the sedative and was opened after consent was obtained. All patients were offered paracetamol and non-steroidal anti-inflammatory drug (ibuprofen or naproxen) before IACI. Eutectic mixture of local anesthetics (EMLA) and cold wrap were applied on each injection site 30 min and 15 min before injection, respectively. Parental presence during IACI was permitted. During the intra-articular corticosteroid injection (IACI), distraction techniques (e.g., videos, music, or audiobooks) could be provided by the parent during administration of either sedative medication.

Patients were continuously monitored with pulse oximetry, ECG and capnometry prongs, and blood pressure was measured every 5 min. Cardiorespiratory parameters, sedation level, and pain scores were followed for 15 min before sedation to establish patient’s baseline.

For treatment under N_2_O sedation, N_2_O was inhaled for 3 to 5 min before the injection. N_2_O was administered with Livopan® consisting of 50% N_2_O and 50% oxygen. Sedation was provided by the pediatric outpatient clinic nurse holding the mask air-tight on the patient’s face. After the procedure the mask was lifted slightly, leading to automatic administration of 100% oxygen. Patient breathed oxygen for 1 min, after which monitoring of vital signs was continued for 2 h.

For treatment under DEX sedation, the patients received i.n. DEX 30 min before the procedure. DEX was administered with MAD Nasal® atomizing device and 1 ml tuberculine syringe [22]. Undiluted 100 µg/ml intravenous concentrate of DEX was administered at a dosage of 4 µg/kg divided equally into both nostrils at a maximum total dose of 100 µg. Patients were followed for 4 h after DEX, or as long as it took them to recover. After the first four patients, the initial dose was reduced to 2 µg/kg due to profound and prolonged sedation observed after the higher initial dose (¾ of the patients receiving 4 µg/kg dose of DEX were observed to have Comfort-B less than 12 at 180 min after administration). If there were no signs of sedative effect within 20 min after DEX, an additional 1 µg/kg dose could be given.

If sedation was inadequate 5 min after N_2_O or 30 min after DEX, oral midazolam (0.5 mg/kg, max. 7.5 mg) was administered to enable the procedure. Need for midazolam was considered a failure of the study drug.

At the next outpatient appointment 1 to 3 months later, the patients and parents responded to a follow-up survey of pain relief and sedation, including open-ended questions on adverse effects. In addition, the preferred sedation for future IACI was asked after the second treatment visit.

### Outcomes

The primary outcome was patient-reported pain assessed with VAS immediately after recovery from each of the two sedation interventions in the crossover design (intranasal dexmedetomidine and nitrous oxide). VAS with a 100 mm line presenting numbers 0–10 and facial expressions reflecting corresponding pain levels was used. VAS below 4.5 was considered mild pain [23]. Secondary outcomes assessing analgosedation were pain VAS assessed by the parent, the pediatric rheumatologist and assisting nurse, and FLACC and Comfort-B assessed by the researcher. Sedation level and pain were assessed every 5 min before, during and 2–4 h after the IACI by using Comfort-B scale and Face, Legs, Activity, Crying, Consolability scale (FLACC) [20, 21]. Adequate sedation on the Comfort-B scale was from 11 to 17, while scores ≤ 10 indicated deep sedation and scores > 17 suboptimal sedation. After the procedure, pain assessment with Visual Analogue Scale (VAS) was collected from the patient, escorting parent, assisting nurse and pediatric rheumatologist performing the procedure.

Heart rate, blood pressure, pulse oximetry, ECG, and respiratory rate by capnometry were monitored as secondary outcomes assessing safety. Pulse oximetry below 92%, blood pressure below fifth percentile (hypotension), and heart rate below two standard deviations from age-appropriate values (bradycardia) requiring intervention were defined as adverse events (AE) [24].

### Statistical analysis

The sample size calculation was based on a previous study reporting mean VAS 3/10 for pain during IACI under N_2_O sedation [11]. Our study hypothesis was that DEX could provide better analgosedation with a mean VAS 2/10. The standard deviation (SD) was estimated to be 2.0. Alpha error 5% and type II error rate 10% were selected, which resulted in 44 children for crossover analysis. As our hospital data from the year 2015 showed 60% of patients requiring IACI only once, the expected total number of patients was 109. Patient enrollment continued until 44 patients were treated with both sedatives.

Two-intervention balanced crossover design was used to compare the primary outcome, patient-reported pain, between N_2_O and DEX sedation. Within-patients treatment comparison was done using linear mixed model where treatment, period, and their interaction as fixed factors and random intercept for subject identifier were included in the model. In addition, group comparisons were made in an unbalanced crossover design including all study participants receiving at least one of the sedatives. Post hoc analysis for VAS score differences between assessors were evaluated using analysis of variance (ANOVA) with Games-Howell correction for multiple comparisons. A linear mixed model for repeated measurements with first-order autoregressive (AR1) covariance structure was used to analyze mean differences between treatments at separate time points for longitudinal hemodynamic parameters and sedation levels. The results of analyses were presented as the means and mean differences with 95% confidence intervals. Data were analyzed using IBM SPSS statistical for Windows software, version 29.0 (IBM Corp, Armonk, NY). Statistical analyses were performed from January 11, 2024 to August 18, 2025.

## Results

**Fig. 1:**
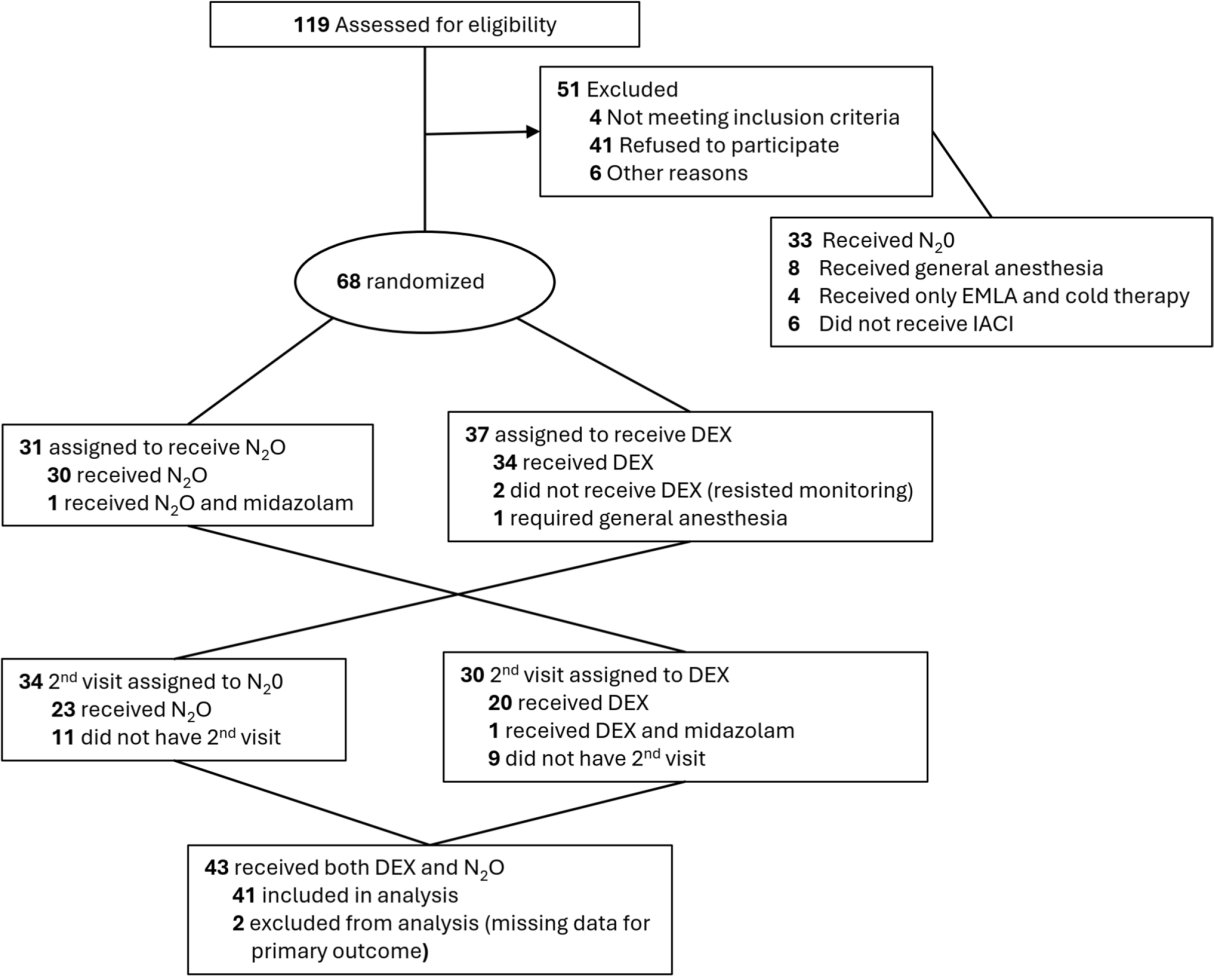
Study design. Balanced crossover analysis for primary outcome was performed on 41 patients due to missing pain VAS from two toddlers. DEX, dexmedetomidine; IACI, intra-articular injection; N_2_O, nitrous oxide; VAS, visual analog scale. Missing data for primary outcome in 2 patients resulted from young age and inability to reliably provide VAS rating

Sixty-eight (57%) of 119 eligible patients, from 2.2 to 16.3 years of age, participated in this trial from February 1, 2017, to December 18, 2023. Seventeen (25%) of the patients were under 6 years of age during the first appointment. Parents of two patients in the DEX group withdrew their consent prior to drug administration because the children resisted monitoring devices. Three patients were excluded from the analysis due to additional sedation requirements prior to injections, two in the DEX group and one in the N_2_O group (Fig. 1). Thirty-one (44%) patients had only one active joint to be treated, and 43% of the patients received their first IACI during the trial. The mean (SD) time interval between the first and second study visit was 7.7 (9.9) months. Detailed patient characteristics are shown in Table 1.

**Table 1:** Demographic data on the study participants at study inclusion

Number of patients		*N* = 68
Sex	Female subjects, *n* (%)	43 (62)
Age at IACI, mean (SD) [range], years		8.8 (3.6) [2.7–16.7]
Duration of the disease, mean (SD), years		1.9 (2.7)
Weight, mean (SD), kg		32.0 (17.2)
Only one inflamed joint, *n* (%)		31 (46)
ICD-10	Diagnosis, *n* (%)	
M08.3	Juvenile polyarhtritis, seronegative	23 (35)
M08.9	Juvenile idiopathic arthritis, unspecified	18 (27)
M08.4	Juvenile oligoarthritis	10 (15)
M13.9	Non-specific arthritis	9 (14)
M08.1	Enthesitis-related juvenile idiopathic arthritis	2 (3)
M08.0	Juvenile polyarthritis, seropositive	2 (3)
M71.2	Synovial cyst of popliteal space [Baker]	1 (2)
L40.5 *M09.0	Juvenile psoriatic arthritis	1 (2)
Previous IACI, *n* (%)		
0		29 (43)
1		7 (10)
2		5 (7)
3		5 (7)
> 3		22 (32)

*IACI* intra-articular corticosteroid injection, *ICD-10* International Statistical Classification of Diseases and Related Health Problems 10th Revision

### Primary outcome

**Fig. 2:**
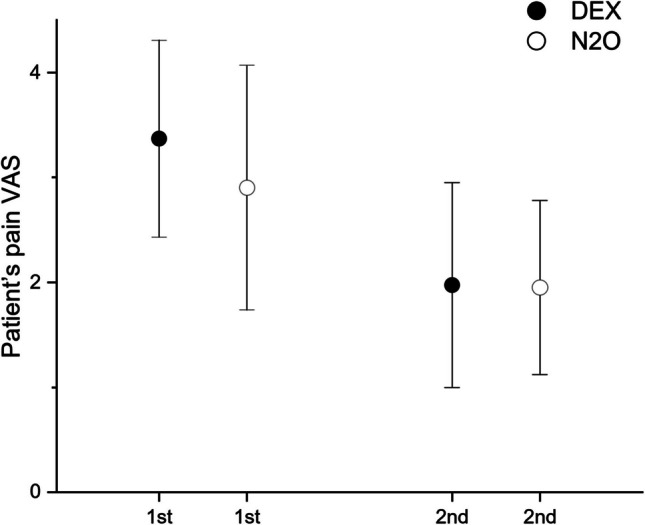
Patient-reported pain during intra-articular injections. Severity of patient-reported pain did not differ between DEX and N2O (difference, 0.2; 95% CI − 0.7 to 1.2, *p* = 0.65). VAS scores were lower at the second appointment, regardless of the medication administered (difference, − 1.2; 95% CI − 2.1 to − 0.2; *p* = 0.02). DEX, dexmedetomidine; N2O, nitrous oxide; VAS, visual analog scale

Severity of patient-reported pain did not differ between DEX and N_2_O in balanced crossover analysis (difference, 0.2; 95% CI − 0.7 to 1.2, *p* = 0.65) immediately after the procedure. VAS scores were lower at the second appointment, regardless of the medication administered (difference, − 1.2; 95% CI − 2.1 to − 0.2; *p* = 0.02). During the first treatment, mean (SD) VAS was 3.4 (2.4) in patients receiving DEX and 2.9 (3.3) receiving N_2_O and at the second treatment, 2.0 (2.1) and 2.0 (1.9) in DEX and N_2_O groups, respectively (Fig. 2).

### Secondary outcomes

Parents observed milder overall pain during nitrous oxide than dexmedetomidine (difference, − 0.9; 95% CI − 1.8 to − 0.04; *p* = 0.04) (Table 2). Mean (SD) VAS for all IACIs during the trial assessed by the patients 2.6 (2.7), accompanying parents 2.8 (2.5) and the assisting nurses 2.4 (2.7) were similar and higher than VAS 1.4 (1.7) assessed by rheumatologists performing the procedures (*p* < 0.05 for all).

**Table 2:**
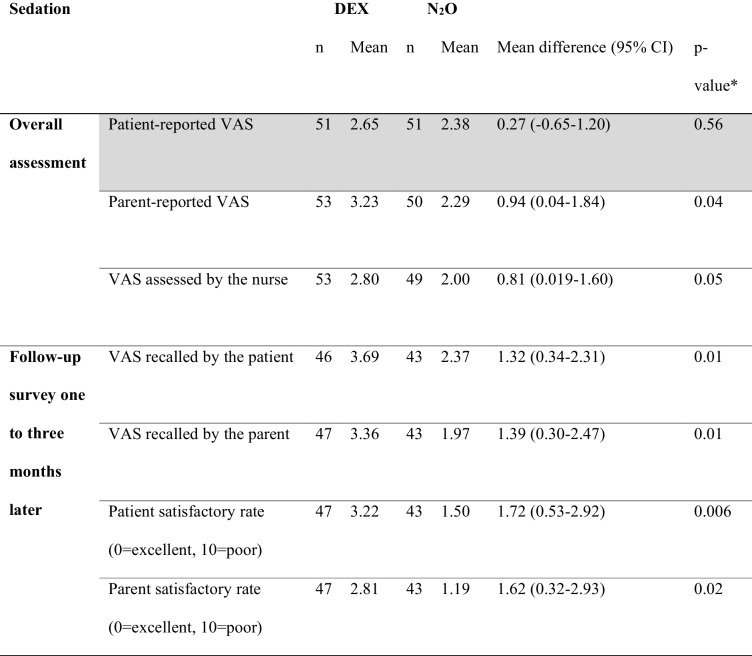
Pain and sedation scores during and after IACIs on i.n. DEX or N2O sedation

*IACI* intra-articular corticosteroid injection, *VAS* visual analog scale for pain, *FLACC* Face, Legs, Activity, Crying, Consolability scale, *Comfort-B* Comfort Behavioural scale

*Unbalanced two-intervention crossover analysis including all treatment visits, also patients receiving only one of the study drugs. The primary outcome measure is shaded in grey for emphasis

In our crossover study, most patients attended two visits, with 1–5 joints injected per visit. The first injection assessed by the rheumatologist and researcher appeared less painful during N_2_O than during DEX (VAS difference, − 0.7; 95% CI − 1.2 to − 0.2; *p* = 0.009, and FLACC difference, − 1.1; 95% CI, − 1.7 to − 0.5, *p* = 0.001). When multiple injections were required during the same visit, pain and sedation scores for subsequent injections did not differ between the groups (Supplementary table S1).

**Fig. 3:**
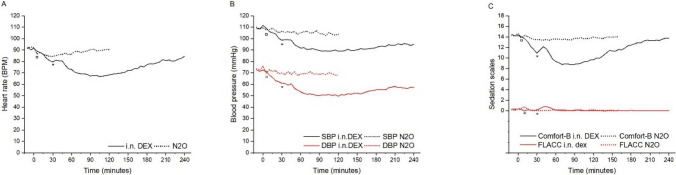
Mean heart rate, blood pressure, and sedation scores. DEX resulted in a more profound and longer-lasting reduction in **A** heart rate 20 min and **B** blood pressure 10 min after drug administrations than N_2_O (*p* < 0.001). **C** Deep sedation (Comfort-B ≤ 10) was commonly observed in DEX group. FLACC did not differ between the groups. DEX, dexmedetomidine; N2O, nitrous oxide; FLACC, Face, Legs, Activity, Crying, Consolability scale

Slight reduction in heart rate was observed during both sedations (Fig. 3a) but was longer lasting and significantly deeper in DEX group after 20 min of drug administration (*p* < 0.001). Relatively profound drop in blood pressure (Fig. 3b) with statistically significant difference between the groups was observed 10 min after DEX (*p* < 0.001).

Suboptimal sedation (Comfort-B scores > 17) at any point in time was observed in 13 (24%) patients in DEX and nine (17%) patients in N_2_O group. Fifty-four (98%) patients receiving DEX experienced deep sedation (Comfort-B 10 or less), compared to six (13%) patients receiving N_2_O. Recovery time from deep sedation to optimal sedation was 53.9 min in DEX and 17.5 min in N_2_O group (mean difference 36.4, 95% CI − 2.4 to 75.2, *p* = 0.07). Rapid decrease in Comfort-B was observed at 20 min and return to optimal sedation level at 135 min after DEX, while sedation effect was lighter in N_2_O group (Fig. 3c). Small increase in Comfort-B and FLACC were observed during IACI in both groups (Fig. 3c).

### Adverse events

Total of 13 AEs occurred; 11 in 54 patients during DEX and two in 53 patients during N_2_O. Hemodynamic AEs (hypotension, bradycardia, asymptomatic 2nd degree AV-block) causing mild unwellness and presyncope were observed in the DEX group. All were easily managed by the Trendelenburg position and tactile stimulation. Other AEs in the DEX group were myoclonic jerks when falling asleep, nausea, and incontinence. Paroxysmal tremor and nausea were observed in the N_2_O group. None of the AEs were serious, required medication, or interfered with the procedure.

### Follow-up

The follow-up survey response rate was 87% (47/54) after DEX and 81% (43/53) after N_2_O. Families reported prolonged sedation in six patients after DEX and in one patient after N_2_O. In addition, lack of sedative effect was reported in one and late onset of sedation after IACI in two patients during DEX. One patient treated with N_2_O reported fear of hospitals, and another had suffered nausea and headache. The majority (69%) of the patients preferred N_2_O for future IACI, 21% DEX, and 10% had no preference.

## Discussion

Both DEX and N_2_O were effective for analgesia and sedation in children undergoing IACI in this randomized prospective crossover trial. The overall subjective pain experience was mild during all injections, with the worst pain occurring during the first appointment. Notable reduction in pain was observed during the second treatment visit in both groups, suggesting that older age and familiarity with the treatment environment appeared to reduce procedure-related anxiety, thereby potentially attenuating the pain experience. This is in line with previously reported findings that sedation during IACI is less commonly required in patients with longstanding JIA compared to those with a recent diagnosis [5].

The pain experience did not differ between the two sedatives at the time of IACIs. As sample size calculation shows study hypothesis was that the initial higher DEX dose, 4 µg/kg, might have provided superior analgesia compared to N_2_O. However, it was considered impractical due to the prolonged and profound sedation it caused. Even with the DEX dose of 2 µg/kg used in the current trial DEX led to deeper and longer-lasting sedation after completion of the procedure. In addition, families reported prolonged sleepiness after DEX more frequently than after N_2_O, reflecting its ability to induce natural sleep-like sedation, if not disturbed [25].

In the context of IACI procedures in children, N₂O was advantageous due to its fast onset, rapid offset, and the ability to finely regulate the duration of sedation through titration of the gas. DEX was delivered as a single intranasal dose via a nasal atomizer, providing an advantage for patients who experience anxiety or fear related to mask‑based administration, but with no possibility to adjust the duration of sedation. Sedation profile with the current DEX dose was inconsistent: sedation was suboptimal in 24% of patients at some point in time during the procedure, followed by deep sedation after the procedure in all but one. The adjustable duration of sedation and quick recovery make N_2_O more attractive option of sedation for short procedures particularly in outpatient care [26].

Pain assessed by rheumatologists and researcher seemed slightly milder during the first injection under N₂O in comparison to DEX, but there was no difference between the drugs during the following injections at the same appointment. In general, rheumatologists assessed the pain milder than patients and parents, while evaluation by the assisting nurse was closer to patient experience. As DEX causes natural, sleep-like sedation, it is quite common that patients woke up momentarily during the IACI, which might result in higher VAS scores assessed by the nurse. Similar finding of researcher and doctor evaluating pain milder than patient have also previously been reported [18, 27] and highlight the importance of multidisciplinary team and family-centered approach, when invasive procedures in pediatric patients are needed.

Despite similar overall VAS during the treatment visit, the patients and their families recalled milder pain and reported higher satisfaction to previous sedation after N_2_O than DEX during the follow-up visit. This may reflect the fact that DEX sedation becomes easily disturbed by painful stimulus, which may provoke a startled reaction, potentially increasing anxiety and leading to a more vivid memory of the pain [28]. Majority of the patients and families preferred N_2_O sedation for future IACIs, which appeared to reflect good analgosedative effect as well as quick recovery from sedation [29]. While N₂O is generally favored, the prolonged facemask use may pose a challenge for patients uncomfortable with masks. In such cases, DEX presents a noninvasive alternative worth considering [30].

The rate of adverse events was low during both study visits, which is in line with previous reports considering both DEX and N_2_O safe and feasible sedatives for children [18]. Cardiorespiratory adverse events occurred more often under DEX sedation, but were easily managed with repositioning and tactile stimulation. Although medications or intravenous fluids were not required, this underscores the importance of continuous patient monitoring when procedural sedation is administered. As dexmedetomidine is contraindicated in patients with second‑ or third‑degree AV block, and one transient AV conduction delay was observed in our study, we suggest that routine ECG monitoring should be performed before and during DEX sedation.

### Strengths and limitations

This patient-centered crossover study, conducted in a real-life pediatric rheumatology outpatient clinic with consecutive and comprehensive recruitment, enabled successful intra-articular corticosteroid injections in patients as young as 2 years. The crossover setting adds robustness of the findings and potentially increase the value of these results for pediatric JIA patients, who often require repeated IACIs as part of their treatment. The dropout rate was low, and the follow-up survey comprehensively collected. The main limitation of this trial was open label format which was inherent in the different administration methods for sedation but reflect a real-life situation. Due to the limited sample size, subgroup analyses by age or joint size were not feasible, although procedural pain may be worse with injections into smaller joints and nitrous oxide may be less effective in younger patients. Furthermore, the reduction of the DEX dose from 4 to 2 µg/kg may have diminished its analgesic efficacy, potentially contributing to the lack of intergroup differences, contrary to the effect anticipated in the sample size calculation. Most patients who were unwilling to participate the study, received N₂O during their IACI.

## Conclusions

Intranasal DEX and N₂O offered effective analgosedation during pediatric IACIs. Both sedatives were safe to use, although mild bradycardia and hypotension were more often observed under DEX sedation. N₂O resulted in better patient experience and became their preference for future sedation.

## Supplementary Information

Below is the link to the electronic supplementary material.

ESM1 Supplementary Table 1 Pain Scores Recorded During Intra‑Articular Injections (DOCX 18.5 KB)

## Data Availability

Deidentified individual participant data will not be made available.

## Abbreviations

AE: Adverse event
DEX: Dexmedetomidine
ECG: Electrocardiogram
EMLA: Eutectic mixture of local anesthetics
FLACC: Face, Legs, Activity, Crying, Consolability scale
IACI: Intra-articular corticosteroid injections
i.n.: Intranasal
JIA: Juvenile idiopathic arthritis
N₂O: Nitrous oxide
VAS: Visual Analogue Scale
