# Supplementary material for: Intranasal dexmedetomidine vs. nitrous oxide for procedural sedation in juvenile idiopathic arthritis (INDEXJIA): a randomized crossover clinical trial
Source: Eur J Pediatr. 2026 Jul 21;185(8):596. doi: 10.1007/s00431-026-07259-w (PMC13388443; doi:10.1007/s00431-026-07259-w)
Supplement: Supplementary file 1 — Supplementary Table 1 Pain Scores Recorded During Intra‑Articular Injections (DOCX 18.5 KB) [file 431_2026_7259_MOESM1_ESM.docx]

**Supplementary table 1** Pain Scores Recorded During Intra‑Articular Injections

| Sedation | | DEX | | N_2_O | |  |  |
| --- | --- | --- | --- | --- | --- | --- | --- |
|  |  | n | Mean | n | Mean | Mean difference (95% CI) | p-value* |
| 1^st^ joint | VAS assessed by the pediatric rheumatologist | 54 | 1.69 | 53 | 1.0 | 0.69 (0.18-1.20) | 0.009 |
|  | FLACC | 54 | 2.41 | 53 | 1.31 | 1.09 (0.45-1.75) | 0.001 |
|  | Comfort-B | 54 | 15.33 | 53 | 14.53 | 0.80 (-0.24-1.84) | 0.13 |
| 2^nd^ Joint | VAS assessed by the pediatric rheumatologist | 36 | 1.60 | 34 | 1.04 | 0.56 (-0.06-1.18) | 0.07 |
|  | FLACC | 36 | 2.56 | 34 | 1.86 | 0.70 (-0.24-1.64) | 0.14 |
|  | Comfort-B | 37 | 15.30 | 35 | 14.96 | 0.35 (-1.13-1.83) | 0.63 |
| 3^rd^ joint | VAS assessed by the pediatric rheumatologist | 18 | 1.36 | 18 | 1.02 | 0.35 (-0.57-1.26) | 0.44 |
|  | FLACC | 18 | 2.49 | 18 | 1.76 | 0.72 (-0.91-2.35) | 0.35 |
|  | Comfort-B | 18 | 16.32 | 18 | 15.67 | 0.66 (-2.66-3.97) | 0.68 |
| 4^th^ joint | VAS assessed by the pediatric rheumatologist | 9 | 1.50 | 12 | 0.66 | 0.84 (-0.29-1.98) | 0.14 |
|  | FLACC | 9 | 2.11 | 12 | 1.38 | 0.74 (-0.97-2.44) | 0.34 |
|  | Comfort-B | 8 | 16.06 | 12 | 15.14 | 0.92 (-1.49-3.33) | 0.39 |
| 5 ^th^ joint | VAS assessed by the pediatric rheumatologist | 4 | 0.83 | 6 | 1.33 | -0.5 (-3.08-2.08) | 0.65 |
|  | FLACC | 4 | 1.50 | 6 | 2.33 | -0.83 (-7.28-5.62) | 0.76 |
|  | Comfort-B | 4 | 14.83 | 6 | 16.00 | -1.17 (-10.40-8.06) | 0.77 |
| VAS; Visual analog scale for pain, FLACC; Face, Legs, Activity, Crying, Consolability scale, Comfort-B; Comfort Behavioural scale  *Unbalanced two-intervention crossover analysis including all treatment visits, also patients receiving only one of the study drugs. | | | | | | | |
